# The functional repertoire contained within the native microbiota of the model nematode *Caenorhabditis elegans*

**DOI:** 10.1038/s41396-019-0504-y

**Published:** 2019-09-04

**Authors:** Johannes Zimmermann, Nancy Obeng, Wentao Yang, Barbara Pees, Carola Petersen, Silvio Waschina, Kohar A. Kissoyan, Jack Aidley, Marc P. Hoeppner, Boyke Bunk, Cathrin Spröer, Matthias Leippe, Katja Dierking, Christoph Kaleta, Hinrich Schulenburg

**Affiliations:** 1 Research Group Medical Systems Biology, Institute of Experimental Medicine, Christian-Albrechts University, Kiel, Germany; 2 Research Group of Evolutionary Ecology and Genetics, Zoological Institute, Christian-Albrechts University, Kiel, Germany; 3 Research Group of Comparative Immunobiology, Zoological Institute, Christian-Albrechts University, Kiel, Germany; 4 Institute of Clinical Molecular Biology, Christian-Albrechts University, Kiel, Germany; 5 Leibniz Institute DSMZ-German Collection of Microorganisms and Cell Cultures, Braunschweig, Germany; 6 Max-Planck Institute for Evolutionary Biology, Ploen, Germany

**Keywords:** Microbiome, Metagenomics

## Abstract

The microbiota is generally assumed to have a substantial influence on the biology of multicellular organisms. The exact functional contributions of the microbes are often unclear and cannot be inferred easily from 16S rRNA genotyping, which is commonly used for taxonomic characterization of bacterial associates. In order to bridge this knowledge gap, we here analyzed the metabolic competences of the native microbiota of the model nematode *Caenorhabditis elegans*. We integrated whole-genome sequences of 77 bacterial microbiota members with metabolic modeling and experimental characterization of bacterial physiology. We found that, as a community, the microbiota can synthesize all essential nutrients for *C. elegans*. Both metabolic models and experimental analyses revealed that nutrient context can influence how bacteria interact within the microbiota. We identified key bacterial traits that are likely to influence the microbe’s ability to colonize *C. elegans* (i.e., the ability of bacteria for pyruvate fermentation to acetoin) and affect nematode fitness (i.e., bacterial competence for hydroxyproline degradation). Considering that the microbiota is usually neglected in *C. elegans* research, the resource presented here will help our understanding of this nematode’s biology in a more natural context. Our integrative approach moreover provides a novel, general framework to characterize microbiota-mediated functions.

## Introduction

Multicellular organisms are continuously associated with microbial communities. The ongoing interactions have likely influenced evolution of the involved microbes and hosts, affecting bacterial growth characteristics or host development, metabolism, immunity, and even behavior [1]. Host organisms and their associated microorganisms (i.e., the microbiota) are thus widely assumed to form a functional unit, the metaorganism, where microbial traits expand host biology [2]. To date, most microbiota studies focus on describing bacterial taxonomic composition, using 16S rRNA amplicon sequencing [3]. These studies revealed that specific taxa reliably associate with certain hosts, for example Bacteroidetes and Firmicutes with humans, *Snodgrassella* and *Gilliamella* with honeybees, or *Lactobacillus* and *Acetobacter* with *Drosophila* [4–6]. 16S profiling, however, is insufficient to identify bacterial functions relevant for the interaction [7]. More insights can be obtained from bacterial genome sequences. For example, genomic analysis of bee microbiota members revealed complementary functions in carbohydrate metabolism, suggesting syntrophic interactions among bacteria [8]. Further, the systems biology approach of constraint-based modeling permits inference of genome-scale metabolic models and prediction of microbial phenotypes [9], as demonstrated for whiteflies and their endosymbionts [10, 11] and also hosts with complex microbiotas [12, 13].

The nematode *Caenorhabditis elegans* is an important model organism in biomedical research. Yet, almost all *C. elegans* research has been without microbiota. In fact, the nematode’s microbiota was only characterized recently, consisting mostly of Gammaproteobacteria (*Enterobacteriaceae*, *Pseudomonaceae*, *Xanthomonodaceae*) and Bacteroidetes (*Sphingobacteriaceae*, *Weeksellaceae*, *Flavobacteriaceae*) [14–17], some of which persist in the worm intestine [15, 18, 19]. The microbiota composition is influenced by both host genotype and environment, and appears similar across geographic regions ([14, 15]; see meta-analysis in [17]). The few studies on microbiota functions highlight an influence on *C. elegans* fitness, stress resistance, and pathogen protection [15]. Previous studies also combined *C. elegans* with soil bacteria, revealing that these can provide specific nutrients [20–24]. Bacterial metabolism can also affect the worm’s response to drugs against cancer and diabetes [25–28]. To date, the functions of the native microbiota have not been systematically explored.

Our aim was to establish the natural *C. elegans* microbiota as a model for studying microbiota functions. We extended previous 16S rRNA data [15] by sequencing whole genomes for 77 bacteria, which are associated with *C. elegans* in nature, most likely as part of the intestinal microbiota, and also *Escherichia coli* OP50, the nematode’s standard laboratory food. We reconstructed metabolic networks from the genomes to explore the bacteria’s metabolic competences and microbe–microbe interactions. We additionally characterized bacterial physiology and assessed which bacterial traits shape colonization ability and influence *C. elegans* fitness.

## Materials and methods

### Materials

Microbiota strains were previously isolated from natural *C. elegans* isolates or corresponding substrates in Northern Germany ([15]; Supplementary Table S1). Briefly, bacteria from worms were obtained after nematodes were thoroughly washed and broken up by vortexing with Zirconium beads. Most bacteria are likely from the intestines, yet an association with the nematode cuticle cannot be excluded [15]. A representative set of 77 strains was chosen for genome sequencing, covering 79.5% of the diversity and abundance of the top ten genera and still 54.6% of that of the top 20 genera from the native *C. elegans* microbiota (some common microbiota members such as Flavobacteria could not yet be isolated [15]). For physiological analysis, bacteria were cultured in tryptic soy broth (TSB) at 28 °C. We performed experiments with the main *C. elegans* laboratory strain N2 (see below), thus allowing us to use previous literature and concurrent experiments with the standard food *E. coli* OP50 as a reference. For these experiments, bacterial TSB cultures (500 µl at OD_600_ = 10) were spread onto peptone-free medium (PFM). Maintenance and bleaching, to obtain gnotobiotic, age-synchronized worms, followed standard methods [29].

### Genome sequencing

Total bacterial DNA was isolated using a cetyl-trimethyl-ammonium-bromid (CTAB) approach [30]. Sequencing was based on Illumina HiSeq and in a subset of nine strains additionally PacBio (Supplementary Table S1). For PacBio, SMRTbell™ template library was prepared following manufacturer’s instructions (Pacific Biosciences, US; Protocol for Greater Than 10 kb Template Preparation). SMRT sequencing was performed on the PacBio *RSII* (Pacific Biosciences, US), applying 240-min movie lengths. PacBio data was assembled using the RS_HGAP_Assembly.3 protocol (SMRT Portal version 2.3.0). Chromosomes and chromids were circularized, unusual redundancies at contig ends and artificial contigs were removed. Error correction was performed by Illumina reads mapping onto genomes using BWA [31] with subsequent variant calling using VarScan [32]. QV60 consensus concordances were confirmed for all genomes. Annotations were obtained with the NCBI Prokaryotic Genome Annotation Pipeline (PGAP). For samples with only Illumina data, low-quality reads and adaptors were trimmed with Trimmomatic v0.36 [33]. Genomes were assembled using SPAdes v3.8.0 [34] and contigs greater than 1000 bp annotated with PGAP and Prokka v1.11 [35]. Genomes were compared with BRIG [36]. BUSCO [37] analysis revealed high-genome completeness, irrespective of sequencing technology (mean completeness of 96.81%; Supplementary Figs. S12 and S13). As assembly quality of the available OP50 genome was low (NCBI project PRJNA41499; >2900 contigs, 73% completeness), we sequenced and assembled it again (218 contigs, 98.7% completeness).

All sequences are available from NCBI Genbank, Bioproject PRJNA400855.

### Reconstruction of metabolic networks

**Fig. 1:**
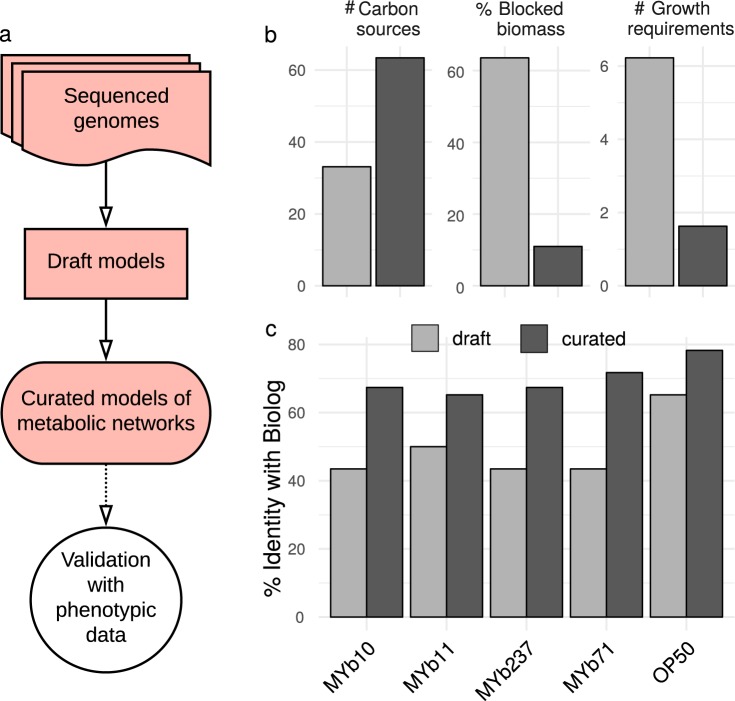
Genomes of bacterial isolates, reconstruction and validation of metabolic networks. **a** Pipeline for metabolic network reconstruction. Sequenced genomes were used to create draft metabolic models. Draft models were curated using topological- and sequenced-based gap-filling. The resulting models were validated with physiological data (BIOLOG GN2; see Fig. 3); these models represent the metabolic networks of microbiome isolates and were used for functional inference. **b** Model improvements by curation, leading to an increase in accurate prediction of uptake of carbon sources, and decreases in the prediction of non-producible biomass components and the number of components needed for growth. **c** Model curation improved agreement with experimental data, as for example the BIOLOG results

Metabolic networks were reconstructed following two steps (Fig. 1a). First, genomes were used to create draft metabolic models, using ModelSEED version 2.0 [38] and associated SEED reaction database. Second, we corrected errors and extended drafts by (i) finding futile cycles, (ii) allowing growth with the isolation medium (TSB), (iii) improving biosynthesis of biomass components, (iv) extending capacities to use different carbon sources, and (v) checking for additional fermentation products. Curation was based on combining topological- and sequenced-based gap-filling using gapseq (version 0.9; https://github.com/jotech/gapseq), pathway definitions of MetaCyc release 22 [39], and UniProt [40]. The presence of enzymatic reactions was inferred by BLAST with bitscore of ≥50 (≥150 for more conservative estimation), and 75% minimum query coverage. Moreover, reactions were assumed to be present if overall pathway completeness was >75% or if it was >66% and key pathway enzymes were present. Host–microbe interaction genes were identified with the virulence factor database [41]. The resulting curated models (Supplementary data S1) were used for further metabolic network analysis. Genome incompleteness did not have a large effect on pathway reconstruction (Supplementary Fig. S14). Computations were done with GNU parallel [42].

### Phylogenetic correlation and clustering of metabolic pathways

We assessed the correlation between metabolic pathway similarity and phylogenetic relationships, using pairwise comparisons of bacteria. We specifically focused on 16S rRNA sequences to calculate phylogenetic similarities, in order to enable comparisons with the standard microbiota analysis approach, based on 16S amplicon sequencing. 16S similarity was scored as percent identity with biostrings [43], using data from the SILVA database [44] based on best hits of extracted genomic 16S rRNA using RNAmmer [45]. To determine overall metabolic distances between isolates, metabolic networks were treated as vectors, clustered horizontally, followed by computation of Euclidean distances between vectors. Cluster similarity was estimated by average linkage and assessed via multi-scale bootstrapping (10,000 replications) using pvclust [46].

### BIOLOG experiments

We used BIOLOG GN2 plates to assess metabolic competences of selected bacteria, including MYb10, MYb11, MYb71, MYb237, and OP50. Bacterial cultures were washed thrice using phosphate-buffered saline (PBS) and density adjusted to OD_600_ = 1.150 µl bacterial suspension per well of BIOLOG plate was incubated at 28 °C for 46 h. Tetrazolium dye absorption (OD_595_) was measured every 30 min (three replicates per strain). Substrate reduction was inferred from fold-change in tetrazolium absorbance:

$${\mathrm{Foldchange}} = \frac{{{\mathrm{OD}}_{t46} - {\mathrm{OD}}_{t0}}}{{{\mathrm{OD}}_{t0}}} - {\mathrm{OD}}_{{\mathrm{control}}}$$

Fold-changes in water were subtracted as background. Hierarchical clustering of strains was based on average fold-change profiles (Ward’s clustering; Euclidean distance) and bootstrapping (*n* = 100). We analyzed metabolic specialization by *k*-means clustering of substrates (*k* = 7, *n* = 10^3^; [47]) (Supplementary Fig. S1). Statistical analyses were performed in R version 3.3.1 [48] and ggplot2 [49].

### Bacterial growth experiments

To validate BIOLOG results, we assessed growth of MYb11, MYb71, and their co-culture in defined media with either alpha-d-glucose or d-(+)-sucrose as carbon sources. We focused on these two isolates, because they are members of two common taxa of the native microbiota of *C. elegans* [15] and because detailed information is available on the interaction of these two isolates with *C. elegans*, including their ability to colonize the nematode gut, persist under stressful conditions, influence nematode population growth, and provide protection against pathogens [15, 18]. Our defined medium is related to S medium [29]: 0.3% NaCl, 1 mM MgSO_4_, 1 mM CaCl_2_, 25 mM KPO_4_, 0.1% NH_4_NO_3_, 0.05 mM EDTA, 0.025 mM FeSO_4_, 0.01 mM MnCl_2_, 0.01 mM ZnSO_4_, 0.01 mM CuSO_4_, and 1% carbon source. Medium without carbon source served as negative and TSB as positive control. Overnight cultures were washed and adjusted to 3.94 × 10^7^ CFUs for growth experiments. Microtiter plates were incubated as BIOLOG plates above. OD_600_ was measured every 30 min, and cultures plated after 48 h. Selective plating of MYb71 using kanamycin (10 µg/ml) allowed to quantify MYb11/MYb71 proportions in co-culture. Three independent runs with technical replicates were assessed with Mann–Whitney *U*-tests and *P*-value adjustment by false discovery rate (fdr, Benjamini Yoav et al. [50]).

### Simulation of bacterial in silico growth

We used the curated models to simulate growth of MYb11 and MYb71 with sucrose as carbon source using BacArena [51]. Sucrose invertases were identified with gapseq (https://github.com/jotech/gapseq) and secreted peptides with SignalP 4.1 [52]. The MYb71 extracellular sucrose invertase was modeled as independent species with a single sucrose invertase reaction and exchange reactions for sucrose, glucose, and fructose. Carbon source utilization and metabolic by-products were predicted using flux balance and variability analysis in R with sybil [53]. Flux balance analysis is a constrained-based method to estimate intra-cellular reaction activities by linear optimization [54], permitting inference of bacterial growth. A carbon source was assumed utilizable if the minimal solution of the corresponding exchange was negative and a byproduct producible if the maximal solution of exchange positive.

### Simulation of ecological interactions

We assessed possible interactions among bacteria using joined models, assuming a common compartment for metabolite exchange between microbes. Activity of individual reactions (i.e., fluxes) was linearly coupled to biomass production to prevent unrealistic exchange fluxes, such as those that solely benefit the partner but not the producer [55]. The objective function was set to maximize the sum of fluxes through both biomass reactions. Two growth media were used for simulations, TSB (Supplementary Table S2) and a glucose minimal medium with thiamine and traces (0.001 mM) of sucrose and methionine to allow initial bacterial growth (Supplementary Table S3). Joined growth rates (j1, j2) were compared to single growth rates (s1, s2). Mutualism was defined as j1 > s1 and j2 > s2, competition as j1 < s1 and simultaneously j2 < s2, parasitism as j1 < s1 and simultaneously j2 > s2 (or j2 < s2 and j1 > s1), and commensalism as j1 = s1 and j2 > s2 (or j2 = s2 and j1 > s1).

### Experimental analysis of bacterial colonization and bacterial effects on *C. elegans* population growth

We examined bacterial colonization of *C. elegans* (i.e., bacteria attached to worms after the washing protocol, thus mainly consisting of intestinal bacteria) by quantifying CFUs extracted from young adults exposed to bacteria for 24 h. In detail, L4 larvae were raised on OP50 lawns and placed on each of the considered bacteria (500 µl, OD_600_ = 10; only one bacterium present). After 24 h, they were washed in a series of buffers (2 × M9 buffer with 25 mM tetramisole, 2 × M9 with 25 mM tetramisole and 100 µg/ml gentamicin, 1 × PBS with 0.025% Triton-X) to remove bacteria from nematode surfaces, and homogenized in the GenoGrinder 2000 using 1 mm zirconia beads (1200 strokes/min, 3 min). Worm homogenate and supernatant control were plated onto tryptic soy agar for quantification.

We further measured worm population growth as a proxy for worm fitness. Three L4, raised on OP50 lawns, were transferred onto lawns with the considered bacteria and total worm numbers counted after five days at 20 °C.

### Regression models

We analyzed the association between phenotypic measurements (i.e., bacterial colonization, worm fitness) and metabolic or virulence characteristics using Spearman rank correlation and random forest regression analysis. Significance of correlations was assessed with permutation tests (100 randomly generated features, FDR-adjusted *P*-values). For random forest regression, the R package VSURF served to select features via permutation-based score of importance [56] and otherwise default settings (ntree = 2000, ntry = p/3).

### Adaptive strategies

According to the universal adaptive strategy theory (UAST) [57, 58], heterotrophic bacteria follow one of three strategies: (i) rapid growth and thus good competitor, (ii) high resistance and thus stress-tolerator, or (iii) fast niche occupation and thus ruderal. We categorized bacterial isolates using published UAST criteria for genomic data [58], which are based on three scores, inferred from genome sequences and metabolic models. In detail, the components of a competitive strategy were large genome size, antibiotics production (presence of pathways belonging to “Antibiotic-Biosynthesis” category in MetaCyc), high-catabolic diversity (Metacyc: “Energy-Metabolism”), and siderophore biosynthesis (Metacyc: “Siderophores-Biosynthesis”). The criteria for stress-tolerators were auxotrophies, slow growth rates in TSB, few rRNA copies, and exopolysaccharides production (MetaCyc pathways: PWY-6773, PWY-6655, PWY-6658, PWY-1001, PWY-6068, PWY-6082, PWY-6073). Those for a ruderal strategy were fast growth in TSB, multiple rRNA copies, and low catabolic diversity (Metacyc: “Energy-Metabolism”). The characteristics of each isolate were related to the other bacteria, yielding a relative score, thereby assuming that different strategies are present in the microbial community as a whole. For each isolate, we assessed whether the inferred value belonged to the lower or upper quantile of this criterium (in case of growth rates we used the mean instead). The total adaptive score per strategy was scaled by the number of features considered per strategy. An isolate was assumed to follow the strategy, for which it produced the highest score. If two strategies had the same score, then we assumed a mixed strategy. The UAST classification remained stable with the same qualitative order, irrespective of genome completeness or sequencing technology (Supplementary Figs. S15 and S16).

## Results

### Genomes of bacterial isolates, reconstruction and validation of metabolic networks

We obtained whole genome sequences for 77 bacterial isolates of the *C. elegans* microbiota (Table 1). Of these, nine were sequenced with PacBio technology, allowing their full assembly, yielding either a single-circular chromosome (four strains) or three chromosomes/chromids in case of the five isolates of the genus *Ochrobactrum*, which is known to have more than one chromosome [59] (Supplementary Table S1). The remaining isolates were sequenced with Illumina only, resulting in assemblies with 11 up to 243 contigs. For four genera (*Ochrobactrum*, *Pseudomonas*, *Arthrobacter*, *Microbacterium*), we included more than five strains and identified substantial intra-generic genome variation (Supplementary Fig. S2).

**Table 1:** Overview of bacterial isolates from the natural microbiota of *C. elegans* included in this study

Phylum	Order	Genus/Family	Isolate
Proteobacteria	Xanthomonadales	*Stenotrophomonas*	MYb238, **MYb57**
Proteobacteria	Pseudomonadales	*Pseudomonas*	MYb1, MYb114, MYb115, MYb117, MYb12, MYb13, MYb16, MYb17, MYb184, MYb185, MYb2, MYb22, MYb3, MYb60, MYb75, **MYb11**, MYb187, **MYb193**
Proteobacteria	Pseudomonadales	*Acinetobacter*	MYb10
Proteobacteria	Enterobacterales	*Erwinia*	MYb121
Proteobacteria	Enterobacterales	*Escherichia*	MYb137, MYb5, OP50
Terrabacteria group	Actinobacteria	*Micrococcaceae*	MYb211, MYb213, MYb214, MYb216, MYb221, MYb222, MYb224, MYb227, MYb229, MYb23, MYb51
Terrabacteria group	Actinobacteria	*Microbacteriaceae*	MYb24, MYb32, MYb40, MYb43, MYb45, MYb50, MYb54, MYb62, MYb64, MYb66, MYb72
FCB group	Bacteroidetes	*Flavobacteriales*	MYb25, MYb44, MYb7
Proteobacteria	Caulobacterales	*Brevundimonas*	MYb31, MYb33, MYb46, MYb52
Terrabacteria group	Bacilli	*Paenibacillaceae*	MYb63
Proteobacteria	Rhizobiales	*Ochrobactrum*	**MYb6**, MYb14, **MYb15**, MYb18, MYb19, MYb29, **MYb49**, **MYb58**, MYb68, **MYb71**, MYb237
Proteobacteria	Burkholderiales	*Achromobacter*	MYb9, **MYb73**
Terrabacteria group	Bacilli	*Bacillaceae*	MYb48, MYb56, MYb67, MYb78, MYb209, MYb212, MYb220
Bacteroidetes	Sphingobacteriales	*Sphingobacterium*	MYb181
Actinobacteria	Actinomycetales	*Rhodococcus*	MYb53

Strains with PacBio sequencing data are given in bold

To study the microbiota’s functional repertoire, we reconstructed genome-scale metabolic models (Fig. 1a and Supplementary Data S1). The initial metabolic models were curated by screening for transporter proteins and filling of missing reactions (gap-filling). Curation increased model quality, including doubling of utilizable carbon sources, reduced absence of essential biosynthesis pathways (e.g., for nucleotides or amino acids) from 60% to below 10%, and reduction in the required additional compounds for growth on defined media from on average six to one (Fig. 1b). To validate our metabolic models, we experimentally quantified the ability of five selected bacteria to utilize 46 carbon sources using the BIOLOG approach. The results produced 49.6% overlap with the initial and 70% overlap with the curated models (Fig. 1c and Supplementary Fig. S9). A 70% overlap is generally consistent with previous studies with model organisms like *Salmonella enterica*, *E. coli*, *Bacillus subtilis*, or *Pseudomonas putida* [60–62]. Notably, the models in these studies were manually reconstructed, highlighting the quality of our automated reconstructions.

### Metabolic diversity within the microbiome of *C. elegans*

**Fig. 2:**
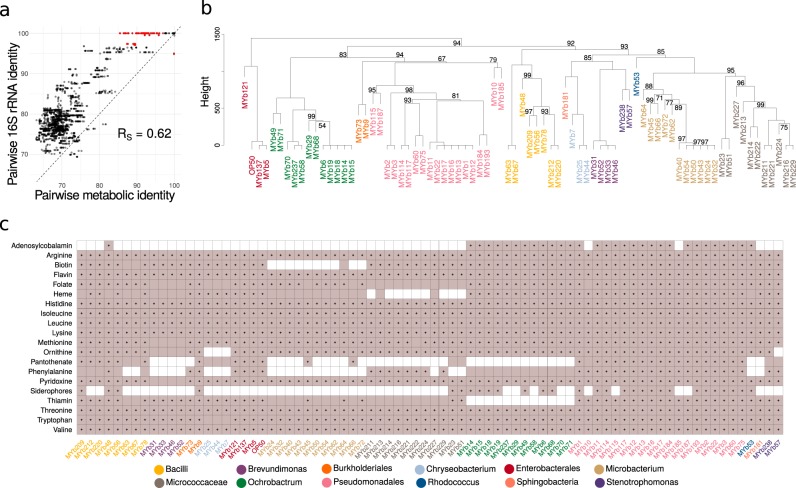
Metabolic network clustering and distribution of important pathways. **a** Correlation between pairwise similarities in 16S rRNA sequences and metabolic networks is shown. Red indicates pairs with a 16S rRNA identity above 97% and metabolic identity below 97% and vice versa. **b** Hierarchical clustering of metabolic networks based on pathway prediction. *P*-values were calculated via multi-scale bootstrap resampling. In case of full support (i.e., *P* *=* 100), *P*-values are not shown (For a complete list of different unbiased *P*-values and bootstrap values see Supplementary Fig. S11). **c** Prediction of bacterial capacity to produce metabolites favoring *C. elegans* growth. Filled squares in light purple indicate that the metabolic networks predict the presence of the biosynthetic pathway required to produce essential amino acids and co-factors. Black dots within the filled squares indicate that pathway presence is supported by more conservative parameters (BLAST bitscore ≥ 150). Different bacterial genera in **b**, **c** are indicated by different colors of the strain names (Table 1)

We used the curated metabolic networks to assess the relationship between metabolic and phylogenetic similarities and the bacteria’s metabolic potential. For phylogenetic relationships, we specifically focused on 16S rRNA sequences, as they are most commonly used to characterize microbiota communities [3]. We found that pairwise 16S phylogenetic relationships are generally indicative of metabolic network similarities (Fig. 2a; Spearman rank correlation, *R*_S_ = 0.6199, *P* *<* 0.0001). Phylogenetic similarities appeared to be larger than metabolic similarities, suggesting some variation in metabolic competences within taxa. Such variation even occurred among isolates with 16S identity above 97%, often used as a species cut-off. This was confirmed through hierarchical clustering of inferred metabolic networks (Fig. 2b), for example for the genus *Pseudomonas* with three clearly separated clusters (see similar patterns for *Enterobacter*, *Ochrobactrum*, or *Microbacterium*). We conclude that variation in metabolic competences is generally related to bacterial phylogeny albeit some variation being present within genera.

We next assessed the bacteria’s metabolic competences (Supplementary Table S4). In general, the inferred metabolic competences are consistent with the aerobic and heterotrophic lifestyle of the *C. elegans* host. The glycolysis, at least the partial pentose phosphate pathway, the tricarboxylic acid cycle, and enzymes enabling oxidative phosphorylation (cytochrome oxidases) were present in all genomes. Almost all isolates possessed enzymes enabling tolerance to microaerobic conditions (e.g., cytochrome bd oxidase). Some Bacilli, *Pseudomonas*, and *Ochrobactrum* showed competences for chemolithotrophic lifestyle (nitrite and formate oxidation) and anaerobic respiration (nitrate, arsenate reduction). Pathways related to CO_2_ fixation (reductive TCA or anaplerosis) were found in several *Pseudomonas*, *Microbacterium*, or Bacilli. Two Bacillales strains showed capacity to degrade polysaccharides, such as starch, cellulose, mannan, rhamnogalacturonan (e.g., *Paenibacillus* MYb63, *Bacillus* MYb67). The microbiota members are able to produce all essential substances required for *C. elegans* growth, which the nematode cannot synthesize on its own (i.e., all essential amino acids and vitamins; Fig. 2c). Most variation among isolates was observed in the biosynthetic pathways of B12, pantothenate, phenylalanine, and siderophores (Fig. 2c). Simulation of in silico growth (Supplementary Fig. S9) suggests that all bacteria can use simple sugars, such as glucose, ribose, or arabinose, while only some can degrade lactose, maltodextrin, or sucrose. Short-chain fatty acids can be generated by all bacteria (Supplementary Fig. S9), while they vary in succinate, cysteine, and valine production. Several microbes possess potential virulence genes, especially *Pseudomonas* and *Escherichia* isolates (Supplementary Table S5).

We subsequently focused on *Ochrobactrum* and *Pseudomonas* isolates. These two genera are enriched in the native microbiota of *C. elegans*, comprising 10–20% of the associated bacteria, they are particularly well able to colonize the nematode gut [15], and some isolates can protect *C. elegans* from pathogens [15, 18]. Most *Pseudomonas* isolates can provide all required substances for nematode growth. *Ochrobactrum* isolates can produce vitamin B12, like *Pseudomonas* isolates, but unlike most other microbiota members (Fig. 2c). Moreover, the *Ochrobactrum* isolates vary from other microbiota members in degradation pathways, energy-metabolism, vitamin biosynthesis, and potential virulence factors (Supplementary Table S6). They apparently lack thiamine and panthothenate vitamin biosynthetic pathways, essential for *C. elegans*. They possess a unique Brucella-like putatively immune-modulating LPS (Supplementary Table S5).

In summary, *C. elegans* harbors a microbial community with diverse metabolic competences, which can supply all essential nutrients for *C. elegans* and includes several *Ochrobactrum* and *Pseudomonas* isolates capable of producing important vitamins such as B12.

### Nutrient context influences ecological interactions within the microbiota

To study how metabolic repertoires affect bacterial growth and interactions within the microbiota, we characterized carbon source utilization of selected isolates and tested growth in different nutrient environments in vitro and in silico. Using the BIOLOG approach, we focused on prominent *C. elegans* microbiota members that colonize worms and affect host fitness, including MYb71, MYb237 (both *Ochrobactrum*), MYb10 (*Acinetobacter*), MYb11 (*Pseudomonas lurida*), and *E. coli* OP50 as control (Supplementary Fig. S3; ref. [15]). For a first insight into bacterial interactions, we additionally included a MYb11-MYb71 mixture (two strains that can co-exist in *C. elegans* [15]).

**Fig. 3:**
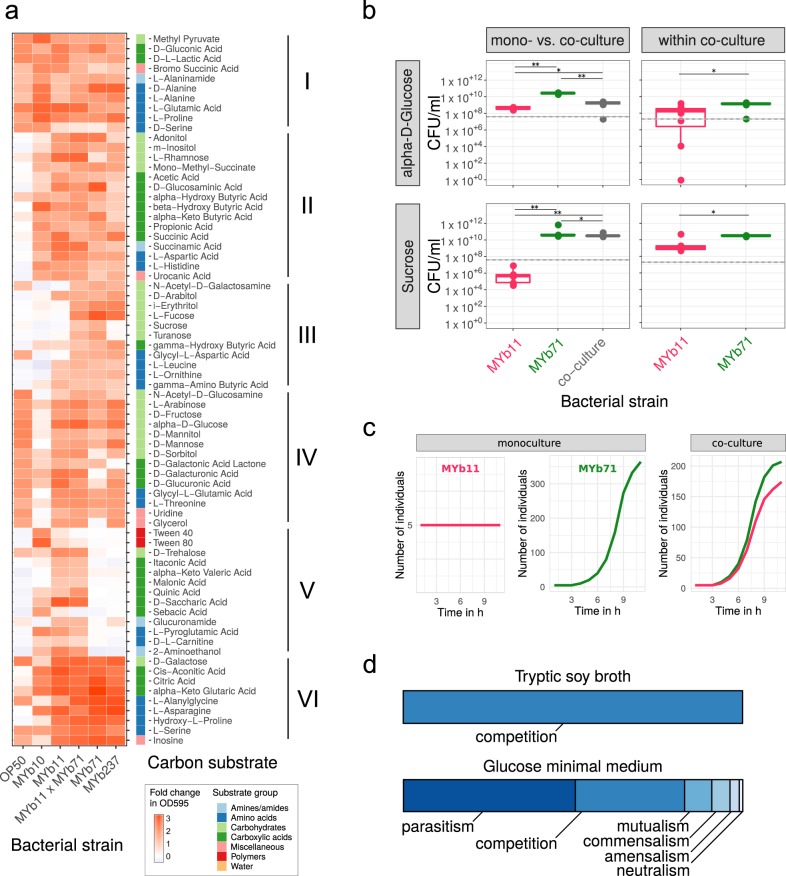
Realized carbon metabolism and growth. **a** Profiles of carbon substrate use of *Acinetobacter* sp. (MYb10), *Pseudomonas lurida* (MYb11), *Ochrobactrum* sp. (MYb71), *Ochrobactrum* sp. (MYb237), and *E. coli* OP50 in BIOLOG GN2 plates over 46 h. The fold-change in indicator dye absorption from 0 to 46 h indicates that the particular compound is metabolized. *k*-means clustering (*k* = 7) of substrates by fold-change highlights metabolic differences between strains. See Supplementary Fig. S5 for cluster VII with substrates used poorly across most strains. **b** Colony-forming units per ml (CFU/ml) of MYb11 and MYb71 in mono- and co-culture at 48 h in alpha-d-glucose and sucrose-containing minimal media. The horizontal and dashed lines indicate mean and SD of CFU/ml at inoculation. Statistical differences were determined using Mann–Whitney *U*-tests and corrected for multiple testing using fdr, where appropriate. Significant differences are indicated by stars (** for *P* *<* 0.01; * for *P* < 0.05). Data from three independent experiments is shown. **c** In silico growth of MYb11 and MYb71 in mono- and co-culture in sucrose-thiamine medium using BacArena with an arena of 20 × 20 and five initial cells per species. **d** Bacterial interaction types observed during in silico co-cultures of all combinations of the 77 microbiota isolates and OP50

Metabolic repertoires of the strains differ and the four microbiota isolates deviate from OP50 in carboxylic and amino acid metabolism (Fig. 3a, cluster II; and Supplementary Fig. S4). MYb10 was least versatile at using carboxylic acids and sugar alcohols (Fig. 3a, cluster IV), while MYb11 and both *Ochrobactrum* could additionally metabolize unique sets of carboxylic acids and sugar alcohols, respectively (Fig. 3a, cluster V and III). Notably, the disaccharides sucrose and turanose were only metabolized by MYb71 (Fig. 3a, cluster III), although sucrose invertases were present in the genomes of both MYb71 and MYb11 (cf. pathway: sucrose degradation I, Supplementary Table S4). In co-culture, the metabolic repertoires of MYb11 and MYb71 appeared additive.

We next assessed whether the differences in MYb11 and MYb71 metabolic competences shape bacterial interactions in growth media with only a single carbon source. We focused on these two isolates as a model and proof-of-principle, because their interaction with *C. elegans* has been characterized in detail, including efficient colonization of nematodes, persistence under stressful conditions, an effect on nematode fitness, and protection against pathogens [15, 18]. We considered growth in the presence of two sugars, which are characteristic for the *C. elegans* natural habitat (e.g., rotting fruits and plant matter). We did not observe any growth in a control medium without carbon source, and thus the tested bacteria are not chemoautotrophic (Supplementary Fig. S6). In minimal medium with alpha-d-glucose, both MYb11 and MYb71 grew, yet exhibited distinct growth dynamics (Fig. 3b and Supplementary Fig. S6). MYb71 produced more CFUs than MYb11 in co-culture (Fig. 3b), suggesting that MYb71 has a growth advantage and/or interferes with MYb11 in some other way. In agreement with the BIOLOG results, a medium including only sucrose supported growth of MYb71 but not MYb11 in monoculture (Fig. 3b and Supplementary Fig. S6). Surprisingly, MYb11 grew in co-culture, indicating parasitic growth (Fig. 3b). Thus, the presence of different carbon sources can change the interaction type between two isolates.

We subsequently assessed the basis for co-growth of MYb11 and MYb71 in sucrose medium, using genome sequence information and in silico growth simulations. Interestingly, we found a secreted sucrose invertase in the genome of MYb71 but not MYb11 (Supplementary Fig. S10). In silico simulations demonstrated that MYb71 can grow in sucrose medium, MYb11 alone does not, while both grow in co-culture (Fig. 3c), confirming our in vitro results. Genome sequence information strongly suggests that growth of both in co-culture is mediated by a secreted enzyme from MYb71.

Taking a more global perspective, we next investigated in silico the potential ecological interactions among bacteria. We compared bacterial growth characteristics in monoculture and co-culture in different nutrient environments. In rich medium (TSB), the exclusive interaction type was competition, indicated by lower growth rates in co- vs. monoculture (Fig. 3d). This changed completely in glucose minimal medium: 50% interactions were parasitic (i.e., the growth rate for one isolate was higher in co-culture than in monoculture, while this pattern was opposite for the other isolate of a pair), 30% were competitive, and 8% mutualistic (i.e., growth rates for both isolates higher in co-culture than the monocultures; Fig. 3d). Under these minimal medium conditions, the most frequently exchanged metabolites across bacteria were glyceraldehyde, acetate, and ethanol (Supplementary Fig. S7). We conclude that the nutrient context modulates bacterial growth, consistently identified in silico and in vitro, and thereby shapes bacteria–bacteria interactions.

### Specific metabolic competences predict bacterial colonization ability and bacterial effects on nematode fitness

**Fig. 4:**
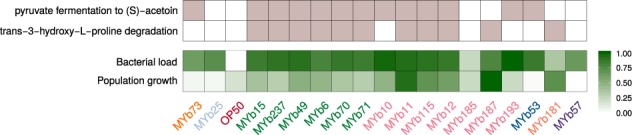
Relationship of bacterial metabolic competences with their colonization ability and their effects on nematode fitness. Presence of metabolic traits (light purple color), which were found to be associated with the bacteria’s ability to colonize *C. elegans* or affect nematode population growth as a proxy for worm fitness (green color). Regression models suggested that the pathway of pyruvate fermentation to acetoin influences bacterial load while the presence of hydroxyproline degradation is associated with *C. elegans* population growth. Colonization and population growth data was normalized; darker colors indicate increased capacities. Different bacterial genera are indicated by the different colors of the strain names (Table 1)

We next characterized traits involved in the interaction between bacteria and *C. elegans*, especially the bacteria’s colonization ability and their effects on worm fitness. We focused on 18 microbiota isolates based on (i) their abundance in the *C. elegans* microbiota, (ii) enrichment in worms, and (iii) effects on worm population growth [15, 63]. OP50 was included as control. The bacteria varied substantially in their ability to colonize *C. elegans* and their effects on nematode fitness (Fig. 4; Supplementary Fig. S3; and Supplementary Table S7). Importantly, these two microbiota characteristics were significantly related with certain metabolic competences. Pyruvate fermentation to (S)-acetoin was significantly associated with bacterial load and the degradation of trans-3-hydroxyproline with nematode population growth (Fig. 4 and Supplementary Table S8).

**Fig. 5:**
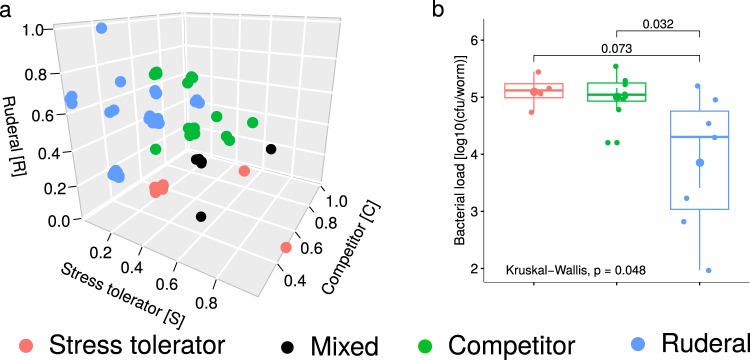
Different adaptive strategies within the microbiota and their relationship to worm colonization. We applied the universal adaptive strategy theory proposed for soil bacteria [58] to categorize the bacterial isolates. **a** Based on genomic and metabolic features, each isolate obtained a score for the competitive (C), stress-tolerating (S), and ruderal (R) strategy, which is represented in the 3D-coordinate system. **b** Bacterial colonization behavior in comparison to adaptive strategies. Isolates that were categorized as ruderal produced the lowest bacterial load, whereas stress-tolerator and competitors had the highest values. The difference in bacterial load between ruderal and other strategies was significant (Wilcoxon rank-sum test, *P* = 0.01)

To further explore the potential behavior of all microbiota isolates in an ecological context, we interpreted their genomic and metabolic traits in light of the universal adaptive strategy theory [57, 58]. Twenty-six isolates were associated with a competitive, nine with a stress-tolerating, and 37 with a ruderal (fast niche occupiers) strategy (Fig. 5a and Supplementary Table S9). The remaining six isolates showed a mixed strategy (same score for competition and stress-tolerance). Interestingly, isolates with different adaptive strategies also varied in their colonization ability (Fig. 5b): bacteria with competitive or stress-tolerance strategies showed higher bacterial load than those with ruderal strategy (Wilcoxon rank-sum test, *P* = 0.01). Moreover, for the competitive and stress-tolerance isolates, bacterial load was significantly correlated with the inferred score (Spearman, *R*_S_ = 0.37, *P* = 0.1; Supplementary Fig. S8). Taken together, the competitive and stress-tolerating strategies are most prevalent within the *C. elegans* microbiota and relate to bacterial colonization capacity.

## Discussion

We here present the first overview of the functional repertoire contained within the native microbiota of *C. elegans* and provide a metabolic framework for functional analysis of host-associated microbial communities. Whole-genome sequences were used to reconstruct the metabolic network of 77 microbiota members. We found that bacterial metabolic competences vary and that the community as a whole can produce nutrients essential for *C. elegans* growth. We identified a significant correlation between metabolic similarities and phylogenetic relationships inferred from 16S rRNA sequences, which are commonly used for bacterial classification. Metabolic variation was larger than evident from 16S data alone, suggesting that metabolic competences can be derived to only limited extent from 16S sequences and should ideally be reconstructed from whole-genome information. For selected bacteria, we validated the model predictions using physiological analyses. Moreover, both in vitro and in silico approaches demonstrated that the nutrient environment can modulate bacterial interactions, for example, from competition to mutualism. We further identified specific metabolic modules that appear to shape the interaction with the host. Finally, we considered a combination of genomic, metabolic, and cellular traits to infer bacterial life history strategies according to the universal adaptive strategy theory [57, 58], finding that bacterial colonization ability is associated with a competitive or stress-tolerant strategy. In the following, we will discuss in more detail (i) the diversity of metabolic competences in the microbiota and possible implications for *C. elegans* biology, (ii) how metabolic networks shape bacteria–bacteria interactions, and (iii) how bacterial traits affect colonization and *C. elegans* fitness.

Our analysis revealed that the microbiota members are jointly able to synthesize all essential nutrients required by *C. elegans*. The considered isolates varied in their capacity to produce vitamins essential to *C. elegans*, such as folate, thiamine, and vitamin B12, which are known to affect nematode physiology and life history [21–23, 25, 64, 65]. For example, vitamin B12 influences propionate breakdown, it can accelerate development, and reduce fertility [21, 65]. Of the characterized bacteria, only *Pseudomonas* and *Ochrobactrum* strains had the pathways to produce vitamin B12. Their enrichment in the microbiota should therefore affect the metabolic state and fitness of *C. elegans*.

Our study demonstrated that the nutrient environment can change bacterial interactions. In our simulations, competitive interactions dominated in rich medium, while parasitic and mutualistic interactions in minimal medium. Interactions between *Pseudomonas lurida* MYb11 and *Ochrobactrum* MYb71 shifted from parasitic to competitive in a sucrose- vs. glucose-supplemented medium. We detected a secreted sucrose invertase in the MYb71 genome, which otherwise lacks sucrose transporters. Thus, we propose that MYb71 breaks down sucrose extracellularly, and the monosaccharides glucose and fructose become exploitable by MYb11. While a similar phenomenon was described for yeast with engineered auxotrophies [66, 67], it was here observed for naturally coexisting host-associated bacteria. This emphasizes the relevance of nutrient context in host microbiota interactions. Importantly, no single growth medium might reliably predict all possible interaction types. It is therefore essential to consider the nutrient context to fully understand bacterial interactions within the microbiota (e.g., ref. [68]).

Our analysis further identified two bacterial traits that appear to influence the interaction with the host. Colonization ability was associated with pyruvate fermentation to (S)-acetoin. This fermentation pathway includes the ketone diacetyl as an intermediate, whose buttery odor attracts *C. elegans* and promotes feeding behavior [69]. In detail, diacetyl binds the transmembrane odor receptor *odr-10* and affects odortaxis [69–71]. As a result, worms are more attracted to bacterial lawns with this particular smell [69]. Indeed, lactic acid bacteria in rotting citrus fruits were more attractive to worms when releasing diacetyl [72]. Similarly, entomopathogenic *Steinernema* nematodes were more attracted to insect cadavers infected with the diacetyl-producing bacterial symbionts of the nematode [73]. Thus, if worms are attracted to diacetyl-producing bacteria, they should spend more time in their presence. This alone could increase bacterial uptake and colonization.

We also found that trans-3-hydroxyproline degradation in bacteria is associated with increased nematode fitness. In worms, hydroxyproline is present in collagen type IV, a major component of the extracellular matrix in the pharynx, intestine, and cuticle [74–76]. The breakdown of hydroxyproline can generate reactive oxygen species [77]. These may act as signaling molecules, which could affect cellular proliferation [78] and *C. elegans* reproduction [79]. Whether ROS in the gut increases brood size is unknown. Alternatively, bacteria with the degradation pathway may utilize the amino acid as a carbon source, consistent with the “microbiome on the leash” hypothesis, characterized by host-selection of beneficial bacterial traits [80].

In conclusion, our study provides a resource of naturally associated bacteria, their whole-genome sequences, and reconstructed metabolic competences that can be exploited to study and understand *C. elegans* in an ecologically meaningful context. This resource may help to further establish *C. elegans* as a model for studying host–microbe interactions.

## Supplementary information

Supplementary Material

Supplementary Table S1

Supplementary Table S2

Supplementary Table S3

Supplementary Table S4

Supplementary Table S5

Supplementary Table S6

Supplementary Table S7

Supplementary Table S8

Supplementary Table S9

Supplementary Data S1
