# Supplementary material for: The functional repertoire contained within the native microbiota of the model nematode Caenorhabditis elegans
Source: ISME J. 2019 Sep 4;14(1):26–38. doi: 10.1038/s41396-019-0504-y (PMC6908608; doi:10.1038/s41396-019-0504-y)
Supplement: Supplementary file 1 — Supplementary Material [file 41396_2019_504_MOESM1_ESM.pdf]

Title

The functional repertoire contained within the native microbiota of the model nematode *Caenorhabditis elegans*

Running title

C. elegans microbiota functions

Authors

Johannes Zimmermann[1*], Nancy Obeng[2*], Wentao Yang[2], Barbara Pees[3], Carola Petersen[2,3], Silvio Waschina[1], Kohar A. Kissoyan[2], Jack Aidley[2], Marc P. Hoeppner[4], Boyke Bunk[5], Cathrin Spröer[5], Matthias Leippe[3], Katja Dierking[2], Christoph Kaleta[1#], Hinrich Schulenburg[2,6#]

Affiliations

1 Research Group Medical Systems Biology, Institute of Experimental Medicine, Christian-Albrechts University, Kiel, Germany

2 Research Group of Evolutionary Ecology and Genetics, Zoological Institute, Christian-Albrechts University, Kiel, Germany

3 Research Group of Comparative Immunobiology, Zoological Institute, Christian-Albrechts University, Kiel, Germany

4 Institute of Clinical Molecular Biology, Christian-Albrechts University, Kiel, Germany

5 Leibniz Institute DSMZ-German Collection of Microorganisms and Cell Cultures, Braunschweig, Germany

6 Max-Planck Institute for Evolutionary Biology, Ploen, Germany

* These authors contributed equally to this work: Shared first authorship

# These authors contributed equally to this work: Shared senior authorship

Correspondence

Christoph Kaleta, Research Group Medical Systems Biology, Institute of Experimental Medicine, Christian-Albrechts University, Michaelisstraße 5, 24105 Kiel, Germany; Tel: +49-431-50030340; Fax: +49-431-50030344; Email: c.kaleta@iem.uni-kiel.de

Hinrich Schulenburg, Research Group of Evolutionary Ecology and Genetics, Zoological Institute, Christian-Albrechts University, Am Botanischen Garten 9, 24118 Kiel, Germany; Tel.: +49-431-8804141; Fax: +49-431-8802403; Email: hschulenburg@zoologie.uni-kiel.de

Conflict of interest

All authors declare no competing financial interests in relation to the work described.

Keywords

Microbiota, *Caenorhabditis elegans*, *Ochrobactrum*, *Pseudomonas*, Metabolic networks

Financial support: German Science Foundation Collaborative Research Center CRC 1182 on Origin and Function of Metaorganisms, projects A1 (KD, ML, HS), A4 (HS), and INF (MPH, CK). Excellence Cluster Precision Medicine in Chronic Inflammation (PMI; CK, HS); the Competence Center for Genome Analysis Kiel (CCGA Kiel; HS); the Max-Planck Society (Fellowship to HS); and the International Max-Planck Research School for Evolutionary Biology (NO).

Supplementary materials

Supplementary figures

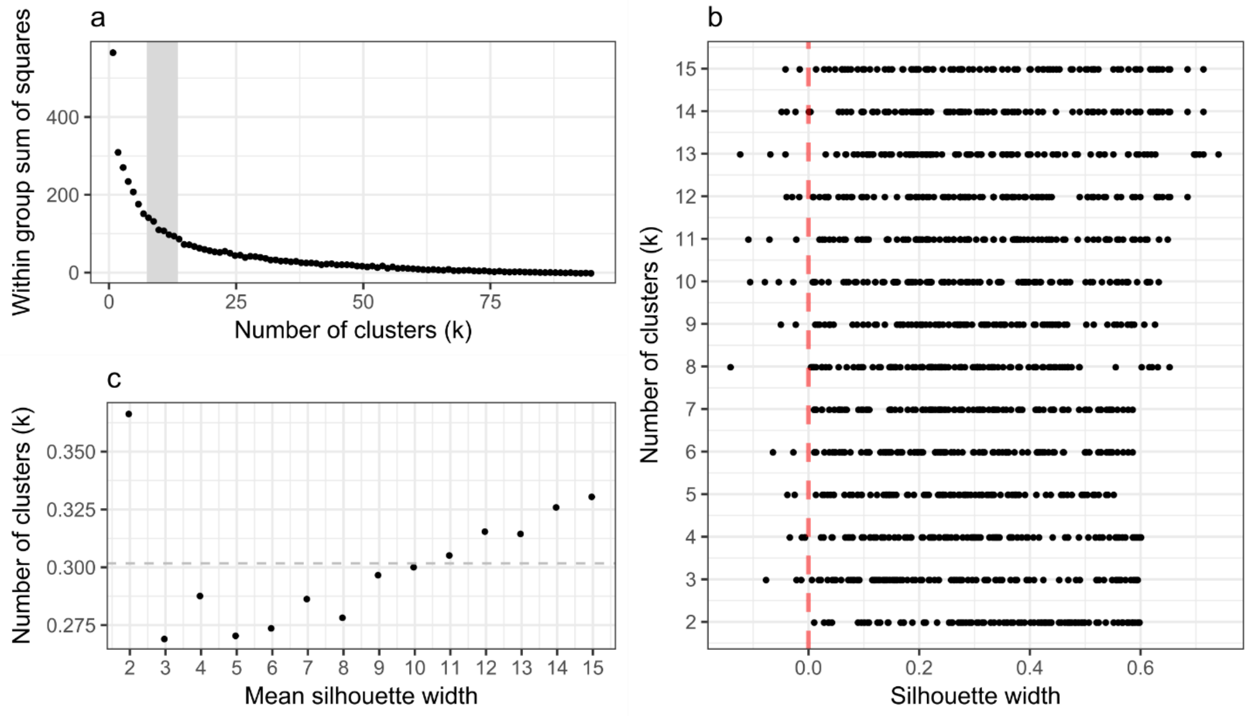

Supplementary Fig. S1. Diagnostic plots of k-means clustering. (a) Within group sum of squares (SS) across clustering with different k. Grey shaded region highlights number of clusters where within group SS starts decreasing less rapidly with an increase in k. (b) Silhouette width of clustering with different k. A silhouette width = 0 indicates points of several clusters overlapping, while a silhouette width = 1 implies a point being exclusive to one cluster. (c) Mean silhouette width across clustering with different k 100 times. Dashed grey line indicates overall mean silhouette width observed.

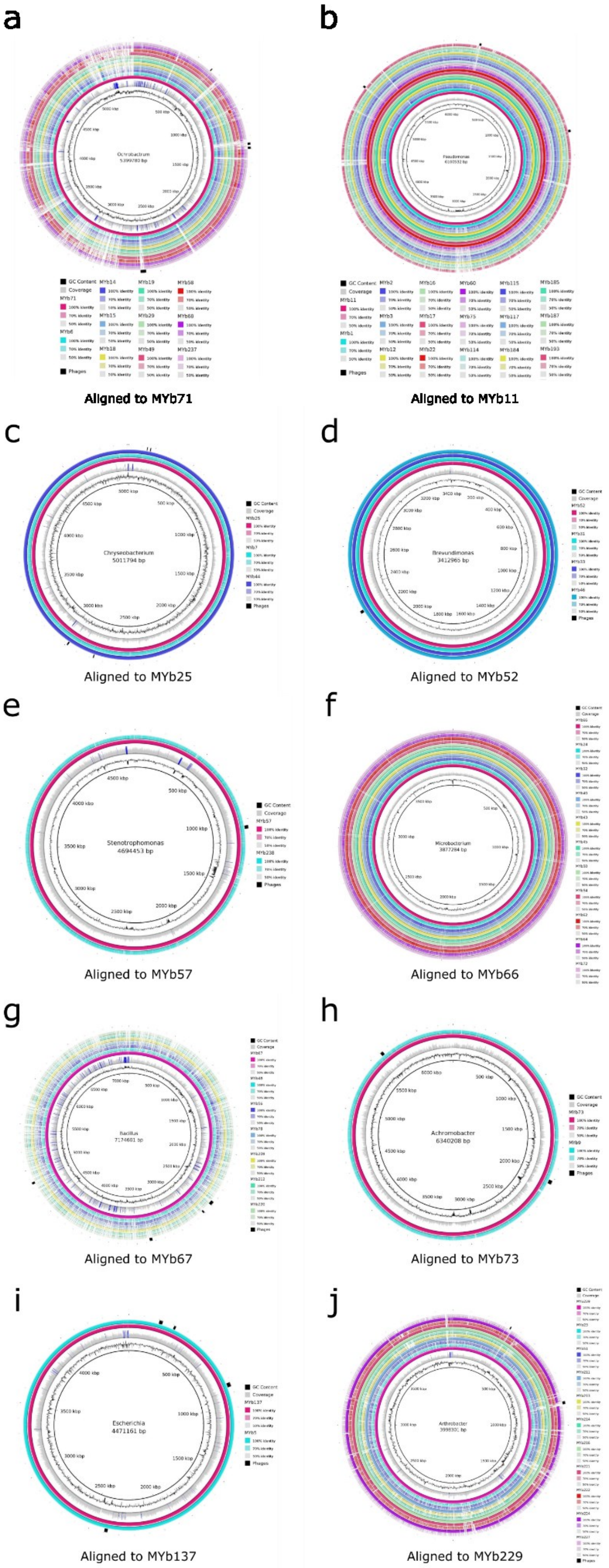
a
b
Aligned to MYb71
Aligned to MYb11
c
d
Aligned to MYb25
Aligned to MYb52
e
f
Aligned to MYb57
Aligned to MYb66
g
h
Aligned to MYb67
Aligned to MYb73
i
j
Aligned to MYb137
Aligned to MYb229

Supplementary Fig. S2. Genome comparison of selected bacterial taxa using circular plots. Selected taxa include: (a) *Ochrobactrum*, (b) *Pseudomonas,* (c) *Chryseobacterium*, (d) *Brevundimonas*, (e) *Stenotrophomonas*, (f) *Microbacterium*, (g) *Bacillus*, (h) *Achromobacter*, (i) *Escherichia*, and (j) *Arthrobacter*. For each taxon, the genome sequence with highest quality (i.e., genome sequence with the smallest number of contigs) was identified and then used as a reference for alignment of the remaining genomes. GC content, coverage, and predicted bacterial phage information are given for the reference genome of each taxon. The color intensity in each ring indicates BLAST match identity. The genomes within a particular taxon are usually highly similar, except in the case of *Bacillus*, which produces higher diversity across the included isolates. Predicted phage regions tend to be highly diverse across isolates within a taxon, except for *Chryseobacterium*, *Brevundimonas*, *Stenotrophomonas*, *Achromobacter*, and *Escherichia*, which may be due to the small number of species in these taxa.

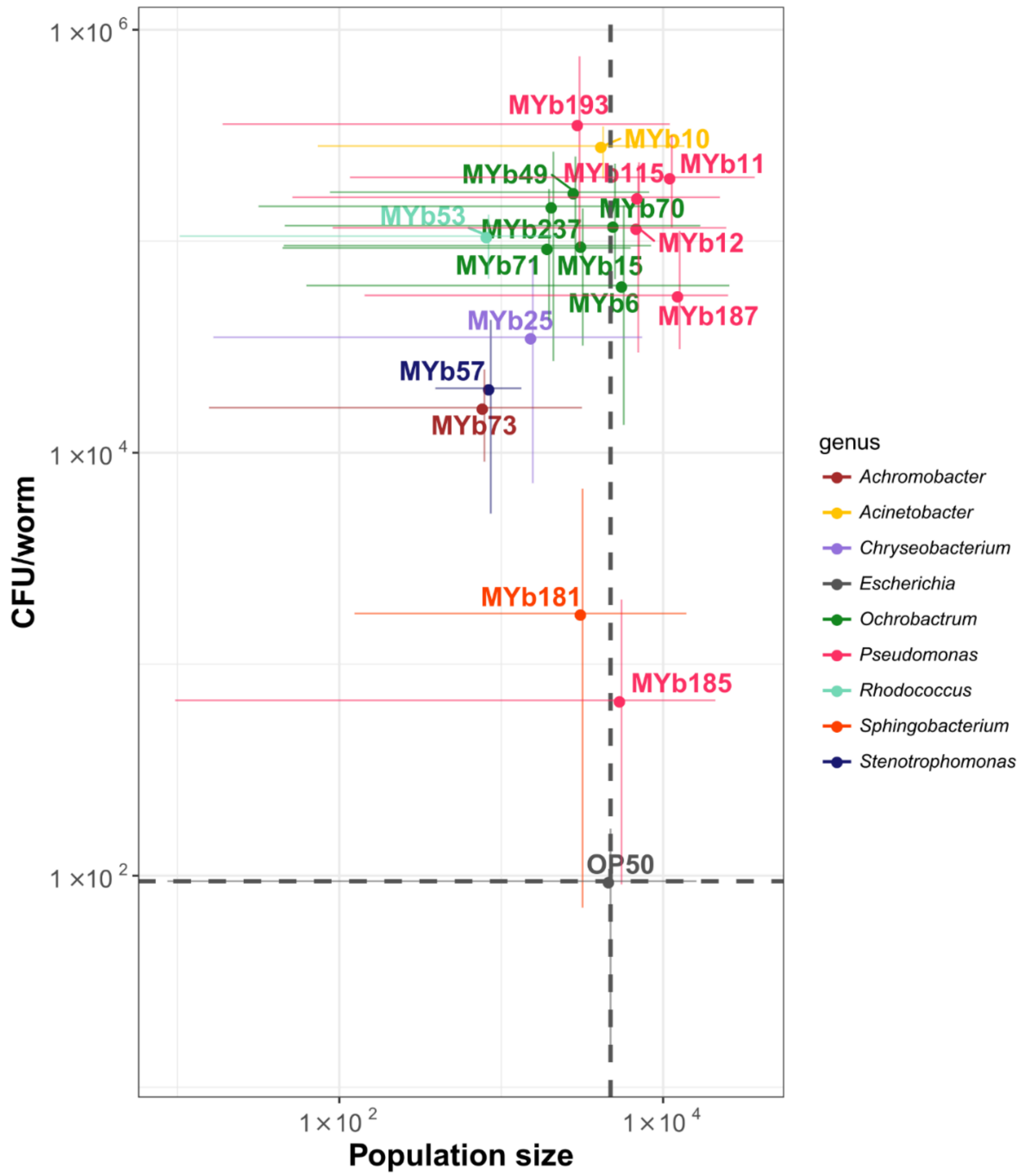

Supplementary Fig S3. Variation in population growth and colonization levels of *C. elegans* in mono-association with natural microbiota isolates. Three L4 larvae were exposed to bacterial lawns on PFM plates for five days, and F2 population sizes quantified (n = 3-6). To count colony forming units per worm, L4 larvae were transferred from NGM plates with OP50, exposed to microbiota lawns for 24 h, washed and the associated bacteria extracted (n = 5). Dashed lines show the mean population size and bacterial load of the canonical food bacteria *E. coli* OP50.

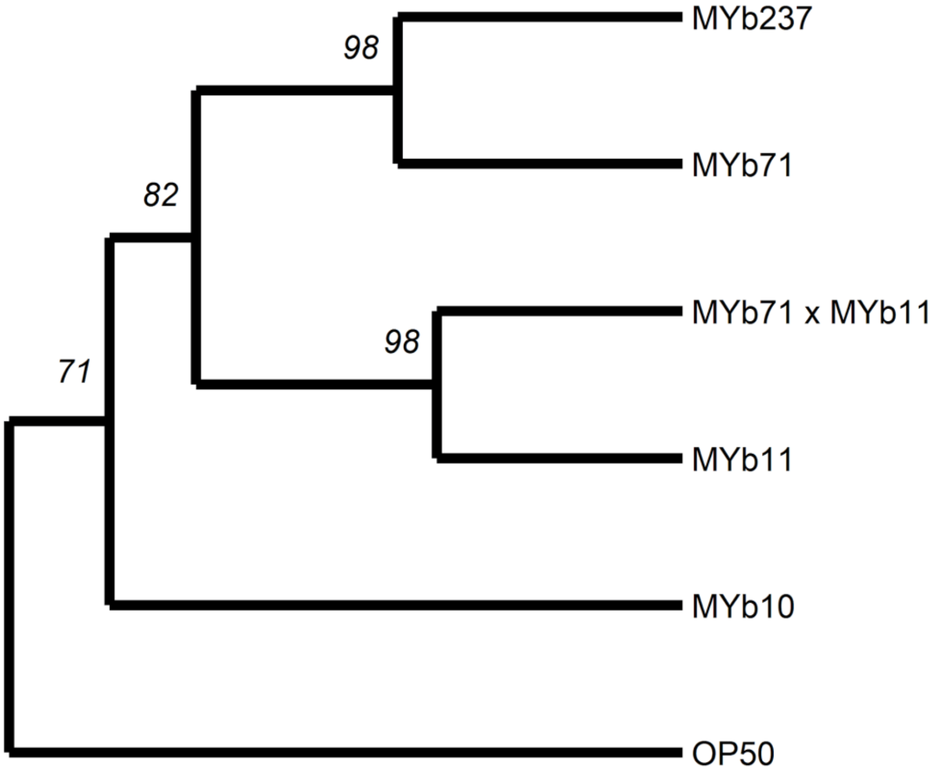

Supplementary Fig. S4. Hierarchical clustering of strains based on BIOLOG profiles. Clustering is based on Ward’s algorithm and Euclidean distance measures. Bootstrap support (nboot = 1000) is shown on nodes.

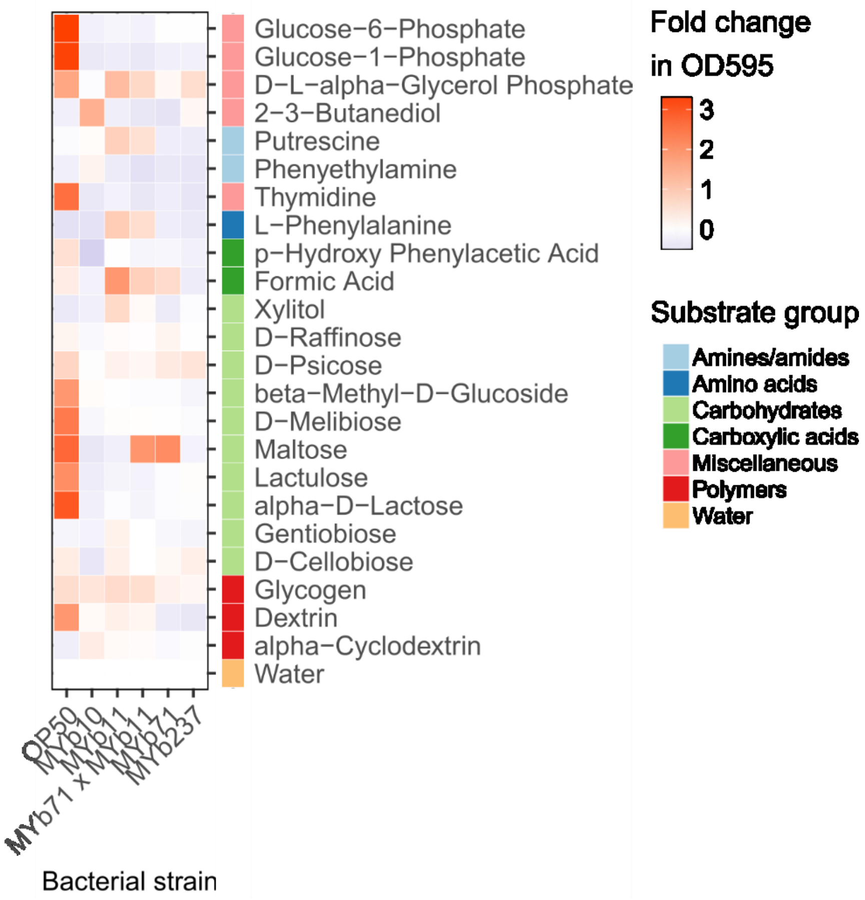

Supplementary Fig. S5. Cluster 7 of BIOLOG profiling. Profiles of carbon substrate use of *Acinetobacter* sp. (MYb10), *Pseudomonas lurida* (MYb11), *Ochrobactrum* sp. (MYb71), *Ochrobactrum* sp. (MYb237), and *E. coli* OP50 in BIOLOG GN2 plates over 46 h. The fold-change in indicator dye absorption from 0 to 46 h indicates that the indicated compound is metabolized. K-means clustering (k = 7) of substrates by fold-change highlights metabolic differences between strains. Clusters I - VI are shown in Figure 3 in the main text.

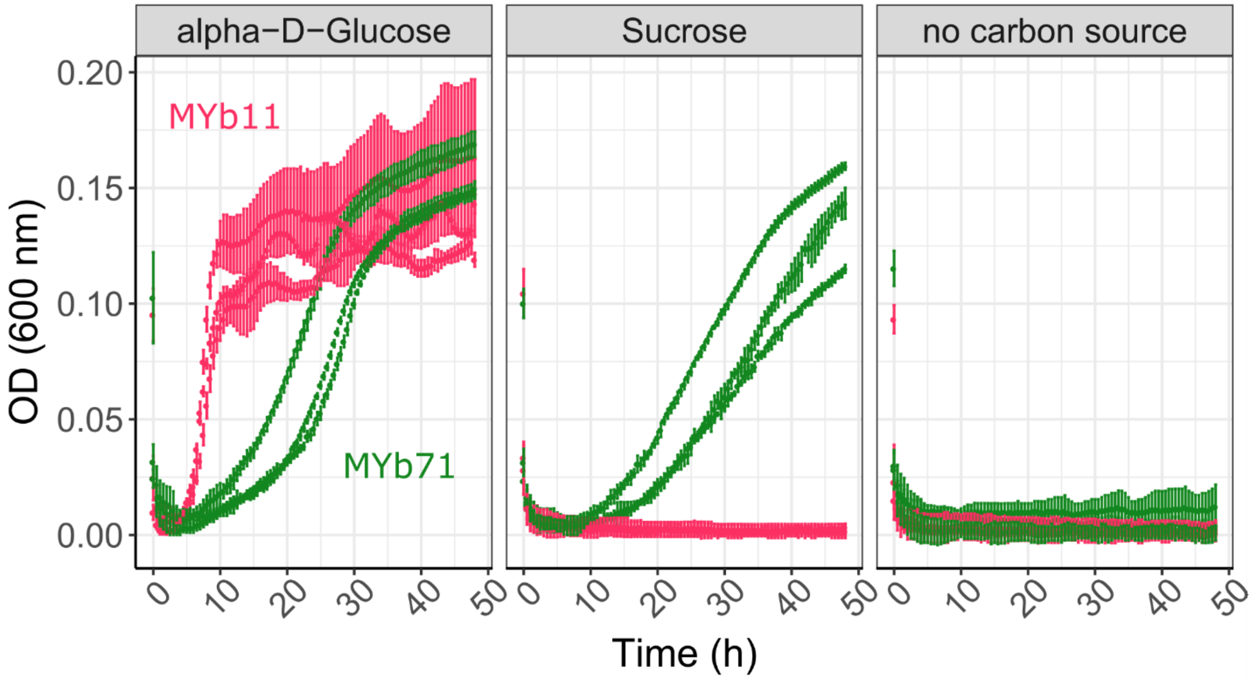

Supplementary Fig. S6. Culture of MYb11 and MYb71 in defined media with single carbon substrates. Growth of MYb11 and MYb71 in chemically defined media with a single carbon source (i.e., alpha-D-glucose or sucrose), or no-carbon control (over 46 h in 96-well plates).

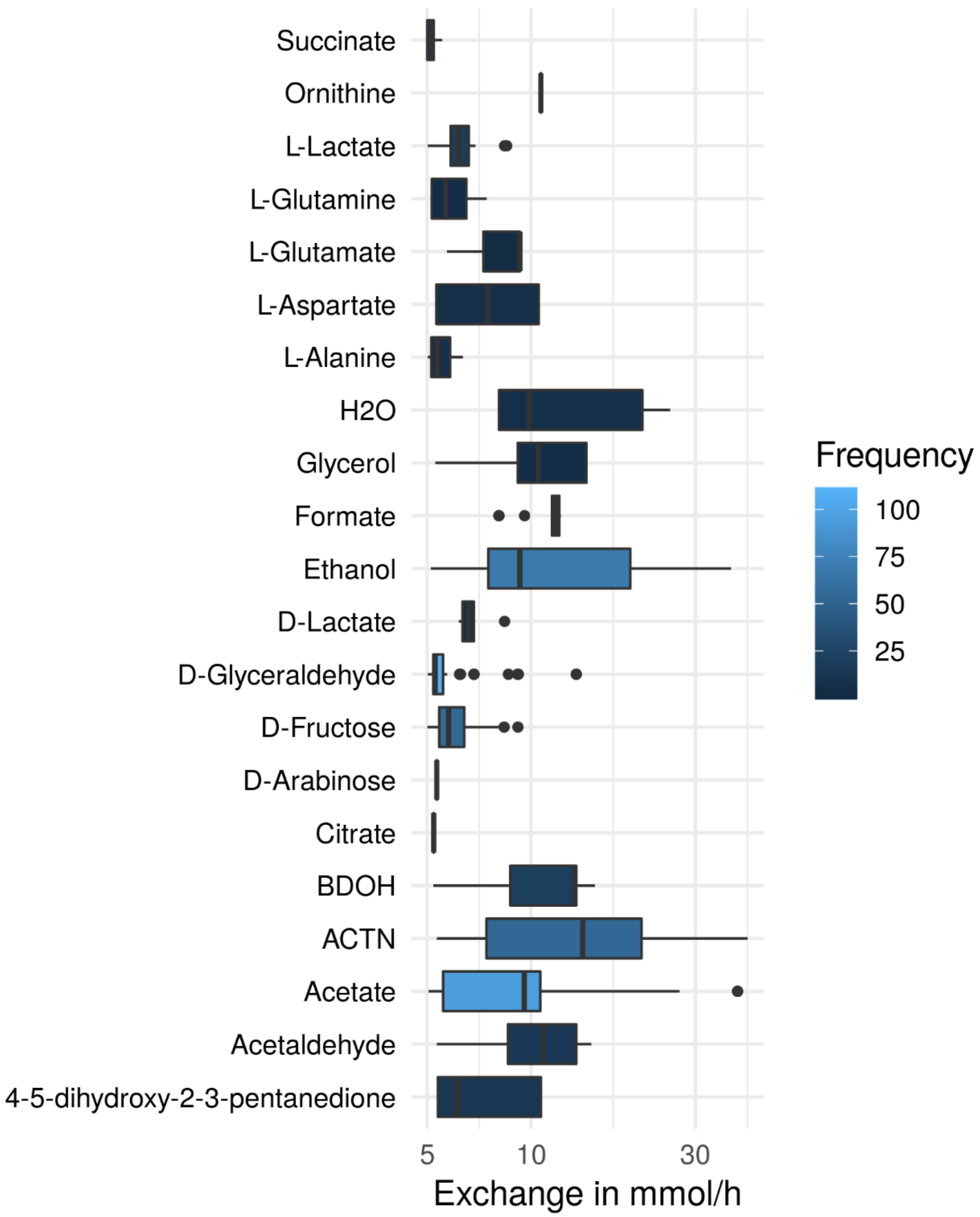

Supplementary Fig. S7. Metabolites exchanged in *in silico* pairwise interactions on a minimal medium. Substances which were exchanged between bacterial isolates in simulation of ecological interactions. In a minimal medium, metabolic byproducts could influence the growth rates of organisms. The figure shows metabolites, which were predicted to be exchanged most frequently and in highest quantity.

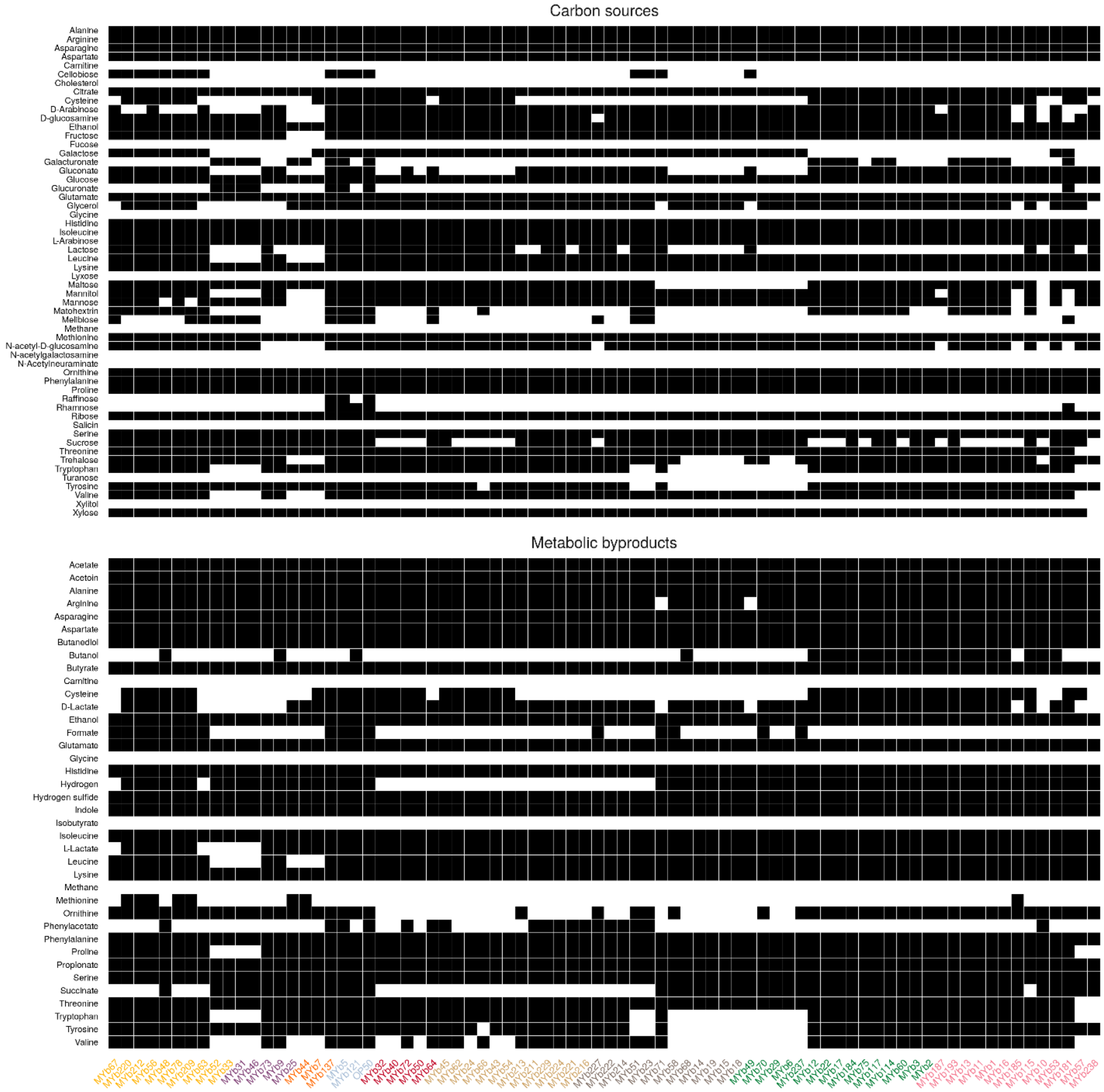

Supplementary Fig. S9. Prediction of carbon sources and metabolic byproducts. In simulation of bacteria metabolism, we predicted substances which could be used as carbon sources (left panel) and substances that may be secreted as metabolic byproducts during for example fermentative processes (right panel). Microbiota isolates are given along the x-axis and compounds along the y-axis.

a

>Sucrose invertase in MYb71 genome

EIHINGGQHV*----YPNKARRLKFSALLCLCLF*SI*QLSCV
FMILCCGESLIDMLPRETAAG--ETAFQPFAGGSVFNTAIA
LGRLDVPTGFFSGISSDFFGEVLRDNLARSNVDYSFAAIS
DRPTT-LAFVRL-VDGQARYAFYDENTAGRMLTESDMPY-
VDDAIDAMLFGCISLISEPCGSVYEALMT-REAPRRVMFL
DPNIRAGFITDREKHLHRMKRMIALADIVKLSDEDLAWF
GEKGSHDEIAAEWLKLGPKLVVITKGAHGADAYTAKATV
RVPGVKVDVVDTVGAGDTVNAGILASLHNQGLLDKDAL
VELTEDQIHSAVALGVRAAAVTVSRAGANPPW

b

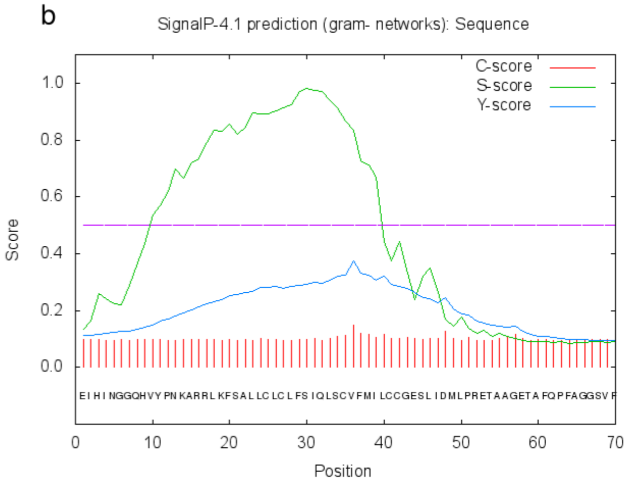

Supplementary Fig. S10. External sucrose invertase in MYb71. (a) Amino acid sequence of the sucrose invertase found in the MYb71 genome. (b) We used SignalP to check for secretory signatures. We found that a discrimination score of D = 0.518 (i.e. weighted average of the mean S and the max. Y scores) which indicates a signal peptide (signalP: Name=Sequence SP='YES' Cleavage site between pos. 35 and 36: LSC-VF D=0.518 D-cutoff=0.420 Networks=SignalP-noTM).

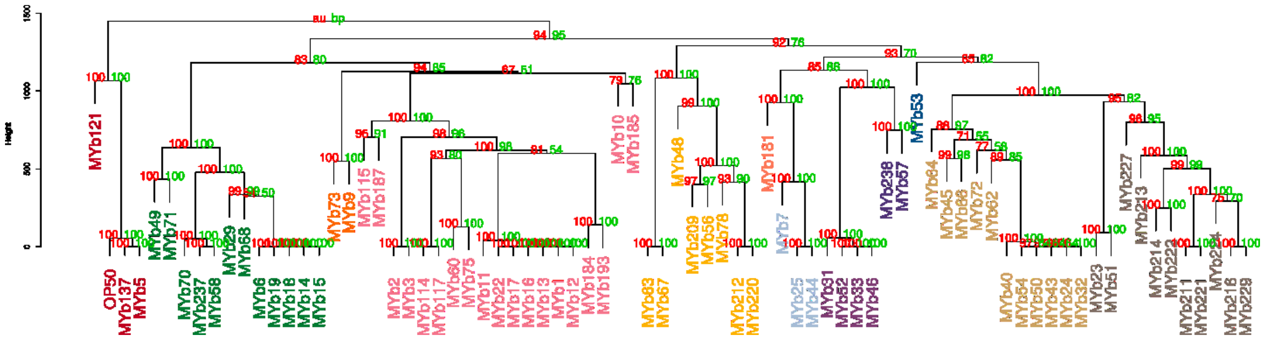

Supplementary Fig. S11. Hierarchical clustering of metabolic networks based on pathway prediction. Metabolic networks were clustered according to their pathway completeness score by Euclidean distances and similarity of clusters was estimated by average linkage. The quality of the clustering was tested by multiscale bootstrap resampling. Green values next to branches indicate the bootstrap probability (number of 90 means e.g. that the cluster exists in 90 of 100 runs). In addition to this, the approximately unbiased p-value from multiscale bootstrap is shown in red (see (1) for details).

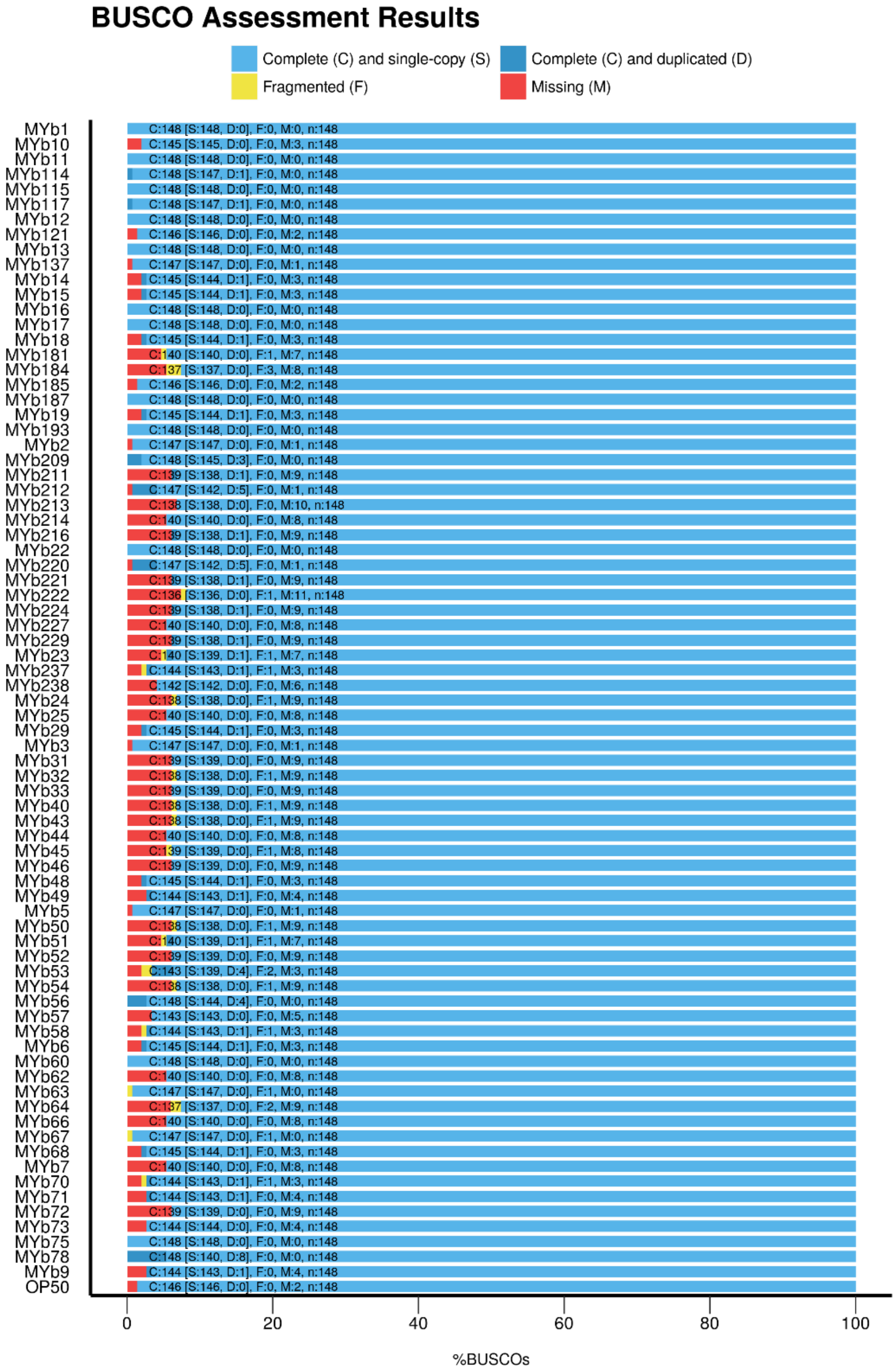

Supplementary Fig. S12. Genomic completeness as assessed with BUSCO. Overview of complete, fragmented and missing matches that were found for all sequenced genomes. The following categories are considered by BUSCO (2): i) complete and single copy: high scoring (i.e.: 90% of the minimum bitscore from an HMM search) and large

alignment length matches with one copy; ii) complete and duplicated: high scoring and large alignment length matches with several copies; iii) fragmented: high scoring but shorter alignment length matches; and iv) missing: low scoring matches. The mean completeness level was 96.81% with a standard deviation of 2.65%. For further details, see the BUSCO user guide (http://gitlab.com/ezlab/busco/raw/master/BUSCO_v3_userguide.pdf).

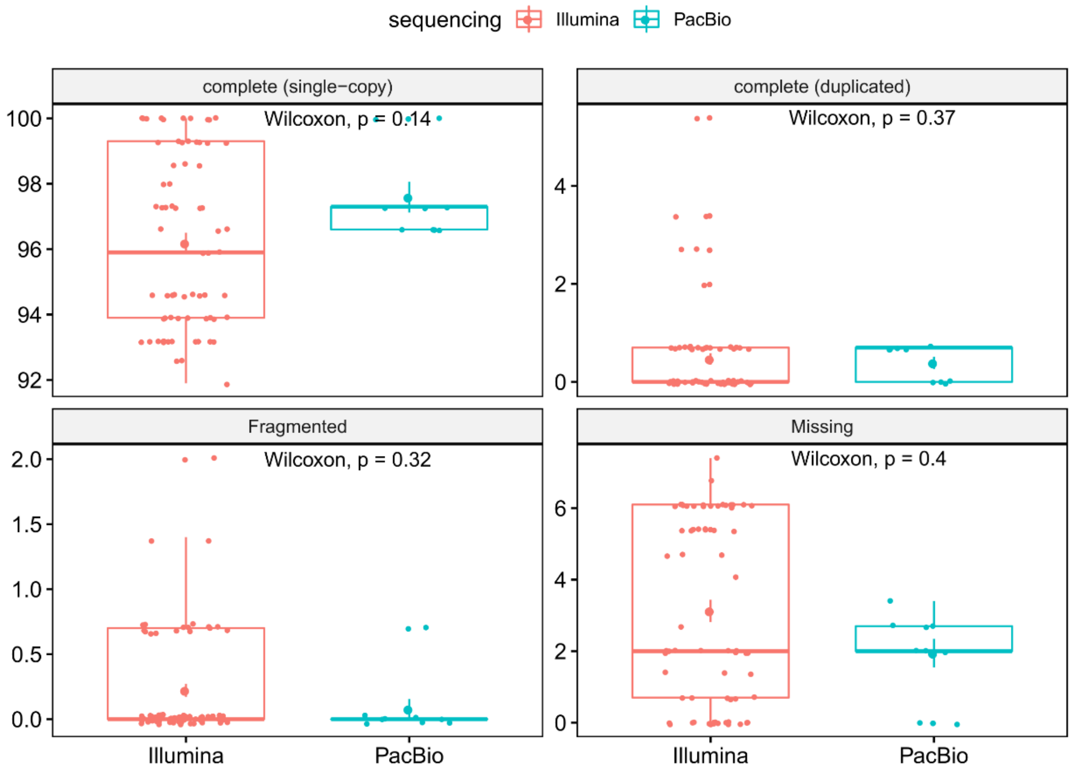

Supplementary Fig. S13. Comparison of genome completeness for different sequencing techniques. Complete, fragmented and missing matches found by BUSCO were compared between genomes reconstructed with either Illumina or PacBio technology, in order to assess to what extent sequencing technique influenced draft genome quality. The four BUSCO categories are the same as those explained in the legend to Supplementary Figure S12. The difference in inferred values between Illumina- and PacBio-sequenced genomes was tested with a Wilcoxon rank sum test. None of the comparisons was significant.

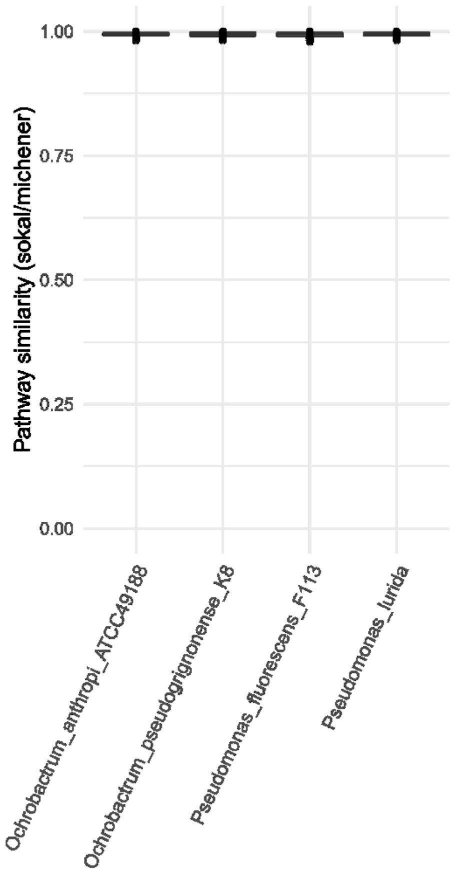

Supplementary Fig. S14. Pathway similarity of incomplete genomes. To assess the influence of incomplete genome coverage on inferred metabolic pathways, we downloaded four high quality genomes of our two focal genera (Pseudomonas and Ochrobactrum) from NCBI. We used the following, published, high quality genomes for this analysis: i) *Ochrobactrum anthropic* ATCC49188 (RefSeq assembly accession numbers: GCF_001652485.1_ASM165248v1); ii) *Ochrobactrum pseudogrignonense* K8 (GCF_000017405.1_ASM1740v1); iii) *Pseudomonas fluorescens* F113 (GCF_000237065.1_ASM23706v1); and iv) *Pseudomonas lurida* (GCF_002966835.1_ASM296683v1). We used these genomes to randomly remove sequence chunks. For this, we simulated similar levels of genome completeness as in our data by drawing from a normally distributed sample parameterized by the results of our

BUSCO genome analysis (mean = 96.81%, sd = 2.65%; Supplementary Figure S12). The presence of metabolic pathways for randomly shortened genomes was compared to the prediction for the original reference genomes. The overlap ranged from 97.8 to 100%. The overall mean pathway similarity between 400 randomly shortened genomes and their complete counterparts was 99.4%.

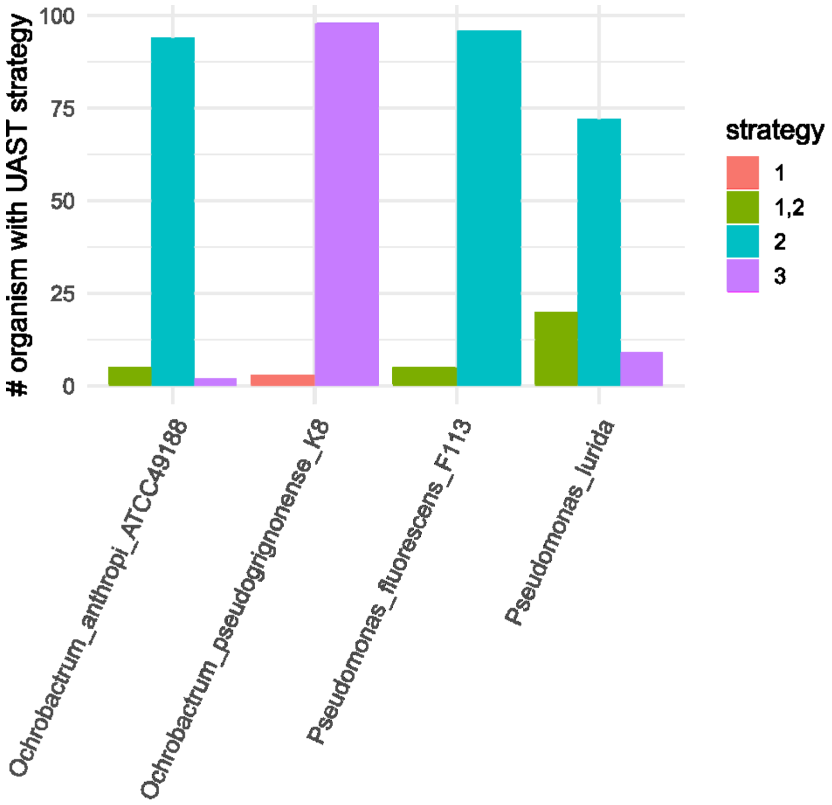

Supplementary Fig. S15. Stability of adaptive strategy predictions. The robustness of UAST classification was tested with randomly shortened representative genomes. High-quality reference genomes were randomly shortened to the same extent as found by BUSCO for the genomes of this study. See legend to Supplementary Figure S14 for more details. For each taxon, 100 shortened genomes were generated and then reclassified according to the universal adaptive strategies (UAST) as done for all other genomes of this study. The bars show how many times a certain strategy was predicted for the incomplete genomes of a particular taxon. The analysis identified the same strategy in at least 94% of shortened genomes for *Ochrobactrum anthropic* ATCC49188, *Ochrobactrum pseudogrignonense* K8, and also *Pseudomonas fluorescens* F113. For *Pseudomonas lurida*, 72% of shortened genomes were consistent with strategy 2 and 20% still with both strategies 1 and 2. A description of the strategies and their definitions is given in Fierer et al. (3).

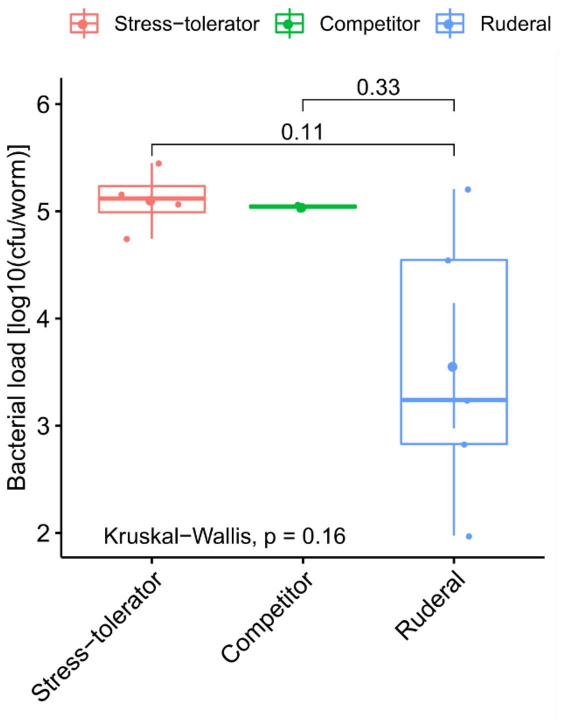

Supplementary Fig. S16. Bacterial colonization behavior in comparison to adaptive strategies for Illumina genomes.

Since sequencing technique could impact rRNA copy number detection, which is used for adaptive strategies classification, we repeated the analysis regarding the colonization potential for Illumina genomes only (69 isolates). Here we found the same qualitative results that competitive and stress-tolerating strategies were associated with higher colonization phenotypic data.

Supplementary tables

Table S1: Genome characteristics. Genomic overview of all bacterial isolates. Based on sequence identity, the closest related species/strains are shown in column B. Genome and assembly statistics (contigs, N50, GC, ...) can be found in columns G:M. The corresponding bioproject to access data via NCBI is shown in column N.

Table S2: *In silico* TSB-based medium. Compounds and maximal uptake rates for Tryptic soy broth (TSB) medium used to simulate bacterial growth.

Table S3: *In silico* glucose minimal medium with thiamine. Compounds and maximal uptake rates for glucose minimal medium with thiamine used to simulate bacterial growth.

Table S4: Predicted pathways. For each bacterial isolate (column A) the predicted presence (column D and column E for with more conservative bitscore cutoff) for all considered metabolic pathways (column C) is given. Here, a number of 1 means present and 0 means not available. In column G the hierarchy (i.e. subsystem) of the pathway is shown.

Table S5: Predicted virulence. In this table the presence of virulence traits based on homology with the virulence factor database is shown. In column A the isolates are given and columns B:AV show the presence of virulence factors (1 for true and 0 for false).

Table S6: Traits with differences in *Ochrobactrum.* Table consists of metabolic pathways and virulence factors which showed to be significantly changed in isolates belonging to the *Ochrobactrum* genus (based on a Wilcoxon signed rank test). For each trait (column B) a FDR corrected P-value (column C) and the mean for Ochrobactrum (column D) and all other isolates (column E) is given.

Table S7: Experimental phenotypic data: bacterial load and *C. elegans* population growth. Sheet 1 shows the number of colony forming units per worm (column D) shown across microbiota isolates (column A) and experimental repetitions of the analysis (i.e., runs; column C). Sheet 2 shows the mean number of worms counted (3 samples counted per population) per population of worms (column C) and standard deviation (column D) on the respective microbiota isolates (column A).

Table S8: Regression analysis to infer metabolic competences associated with bacterial colonization and host fitness. Two regression approaches were used to find traits which were associated with experimental data of bacterial load in *C. elegans* and the fitness of *C. elegans* when the worm was grown together with the bacterial isolates. In this table, the significant/important traits of both approaches are listed.

Table S9: Traits and scores used to categorize isolates according to adaptive strategies. Table of metabolic traits and model features associated with stress-tolerating, competitive, or ruderal strategies. For each isolate the scores and classification for each strategy are listed.

Supplementary data

Supplementary data S1. Genome-scale metabolic models of the microbiota of C. elegans. Zip archive of metabolic models for each isolate in Systems biology markup language (SBML) format. In addition to this, an R file with a list of all models is provided.

Supplementary bibliography

1. Suzuki R, Shimodaira H. Pvclust: an R package for assessing the uncertainty in hierarchical clustering. Bioinformatics. 22:1540–2.
2. Simão FA, Waterhouse RM, Ioannidis P, Kriventseva EV, Zdobnov EM. BUSCO: assessing genome assembly and annotation completeness with single-copy orthologs. Bioinformatics. 201;31(19).
3. Fierer N. Embracing the unknown: disentangling the complexities of the soil microbiome. Nat Rev Microbiol. 2017;15(10):579–90.
